# Artificial reef design affects benthic secondary productivity and provision of functional habitat

**DOI:** 10.1002/ece3.6047

**Published:** 2020-02-08

**Authors:** Sally Rouse, Joanne S. Porter, Thomas A. Wilding

**Affiliations:** ^1^ Scottish Association for Marine Science Oban UK; ^2^ International Centre for Island Technology Heriot Watt University Orkney Stromness UK

**Keywords:** artificial reef, epifauna, functional habitat, reef design, secondary productivity

## Abstract

1. Novel hard substratum, introduced through offshore developments, can provide habitat for marine species and thereby function as an artificial reef. To predict the ecological consequences of deploying offshore infrastructure, and sustainably manage the installation of new structures, interactions between artificial reefs and marine ecosystem functions and services must be understood. This requires quantitative data on the relationships between secondary productivity and artificial reef design, across all trophic levels. Benthic secondary productivity is, however, one of the least studied processes on artificial reefs.
2. In this study, we show that productivity rates of a common suspension feeder, *Flustra foliacea* (Linnaeus 1758), were 2.4 times higher on artificial reefs constructed from “complex” blocks than on reefs constructed from “simple” blocks, which had a smaller surface area.
3. Productivity rates were highest on external areas of reefs. Productivity rates decreased by 1.56%, per cm distance into the reef on complex reefs and 2.93% per cm into the reef on simple block reefs. The differences in productivity rates between reefs constructed from simple and complex blocks are assumed to reflect different current regimes and food supply between the external and internal reef areas, according to reef type.
4. *Synthesis and applications.* Our results show that artificial reef design can affect secondary productivity at low trophic levels. We demonstrate that the incorporation of voids into reef blocks can lead to a greater proportion of the structure serving as functional habitat for benthic species. By including such modifications into the design of artificial reefs, it may be possible to increase the overall productivity capacity of artificial structures.

## Graphical abstract

Artificial reef design can affect secondary productivity of organism low trophic levels. We demonstrate that the incorporation of voids into reef blocks can lead to a greater proportion of the structure serving as functional habitat for benthic species, and a higher overall productive output for the reef.

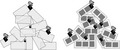

## 1 INTRODUCTION

Structures are introduced into marine environments for coastal protection, food production, dive tourism, oil and gas extraction, and, more recently to support renewable energy generation. The ecological consequences of the wide‐scale deployment of artificial structures in marine environments, termed “ocean sprawl,” has been identified as a priority for research (Firth et al., 2016). When artificial hard substrates are introduced into marine habitats, they are rapidly colonized by sessile epifauna and flora. Subsequently, a community of mobile species develops that include invertebrates, fishes, and marine mammals. (Aseltine‐Neilson, Bernstein, Palmer‐Zwahlen, Riege, & Smith, 1999; Miller, 2002; Miller et al., 2013; Vaissière, Levrel, Pioch, & Carlier, 2014). These “artificial reef” communities support ecosystem service delivery, which can include water filtration, carbon sequestration, and the production of commercially exploitable biomass (Dafforn et al., 2015; Moberg & Rönnbäck, 2003).

The effects of artificial reef structures are frequently viewed as positive environmental change, or as mitigation against negative consequences that may arise from marine developments (Gill, 2005). Negative consequences of marine developments can include alteration of sedimentary habitat underneath a structure (Heery et al., 2017) or from a socioeconomic perspective, loss of seabed access for commercial fishers (Alexander, Potts, & Wilding, 2013; Miller et al., 2013). In order to accurately predict the effects of artificial structures, or the degree to which structures can deliver the desired mitigation (e.g., biomass exportation of commercial fish species), it is necessary to understand the development and functioning of artificial reef communities. Colonization and successional patterns of artificial reef communities have been studied on shipwrecks, oil platforms, and coastal defense structures (Firth et al., 2014; Gallaway, Szedlmayer, & Gazey, 2009; Husebø, Nøttestad, Fosså, Furevik, & Jørgensen, 2002; Pickering, Whitmarsh, & Jensen, 1999). However, much uncertainty remains over the local and regional benefits of artificial reefs, and the mechanisms that regulate ecosystem functioning and services on artificial reefs. This is particularly true in temperate marine systems, which have received comparatively little attention in terms of artificial reef research (Jensen, 2002).

Secondary productivity, which describes the rate of biomass production by heterotrophic organisms, is one of the most commonly used measures of ecosystem functioning (Dolbeth, Cusson, Sousa, & Pardal, 2012; Hooper et al., 2005). Measures of secondary productivity indicate the energy flow through food webs and serve as a useful metric for comparing organisms and ecosystems that support different life histories and assemblages (Dolbeth et al., 2012). Previous research on artificial reef secondary productivity has largely focused on fish and has showed that in some cases artificial structures have the potential to support higher secondary productivity than natural reef habitats (Claisse et al., 2014; Cresson et al., 2019).

The sessile benthic components of artificial reef communities are an integral part of the local ecosystem and temperate assemblages typically comprise a range of hydroids, bryozoan, sponges, and anemones (Van der Stap, Coolen, & Lindeboom, 2016). Through trophic linkages, the secondary productivity of benthic epifauna supports productivity of species higher in the food chain, including commercially important species. Epifaunal productivity is, however, one of the least investigated aspects of artificial reef ecology (Becker, Taylor, Folpp, & Lowry, 2018; Lima, Zalmon, & Love, 2019; Moura, Fonseca, Boaventura, Santos, & Monteiro, 2011). Quantifying epifaunal productivity requires data on the spatial variability in epifaunal growth and reproduction rates on structures, according to both the design of the structure and the receiving environment into which the structure is placed.

Artificial reef design determines the level of habitat complexity offered to marine organisms. Habitat complexity can be considered as a function of the rugosity of materials, the presence of crevices/holes, the configuration of reef component parts, and the design of the individual reef blocks/components themselves (for reefs constructed from multiple parts) (Loke & Todd, 2016). Variations in habitat complexity can manifest as differences in the available surface area and/or the variety and type of structural components, termed “structural diversity” (Loke & Todd, 2016). On both natural and artificial reefs, habitat complexity is known to be positively correlated with the diversity and abundance of species (Charbonnel, Serre, Ruitton, Harmelin, & Jensen, 2002; Hunter & Sayer, 2009). However, the effects of habitat complexity on reef fauna are scale dependent (Dahl, 1973; Frost, Burrows, Johnson, Hanley, & Hawkins, 2005; Wilding, Rose, & Downie, 2007), and species will respond to different scales of habitat complexity according to their body size and habitat requirements (McAbendroth, Ramsay, Foggo, Rundle, & Bilton, 2005).

In addition to determining habitat complexity, the design of an artificial reef will affect near‐reef physical conditions, including the current regime and sedimentation. For filter‐feeding epifauna, near‐reef flows and boundary‐layer conditions will influence food supply, their ability to attach to the substrate and sediment resuspension and smothering (Wildish & Kristmanson, 2005). These near‐reef physical conditions dictate the extent to which different areas of the reef serve as functional habitat for epifauna species and, ultimately, the total productive output of the reef. In order to predict the functional habitat provision from specific structure designs and develop models of the actual or potential productive capacity of a reef, quantitative data on the relationship between epifaunal productivity and reef design are required (Aseltine‐Neilson et al., 1999).

The aim of this study was to use transplants of the common epifaunal suspension feeder *Flustra foliacea* (Linnaeaus 1758) to quantify secondary productivity on two different designs of artificial reef and use this information to compare how different reef designs affect the provision of functional habitat for epifauna.

## 2 MATERIALS AND METHODS

### 2.1 Experimental approach

This study used a SCUBA diving approach to transplant fronds of *F. foliacea* to internal and external areas of six artificial reef modules with differing designs. The growth rates of transplanted fronds were measured to quantify the relationship between secondary productivity and distance into the reef as a function of reef design, and to compare the availability of potentially functional habitat for suspension feeders between reef designs.

### 2.2 Study site

**Figure 1:**
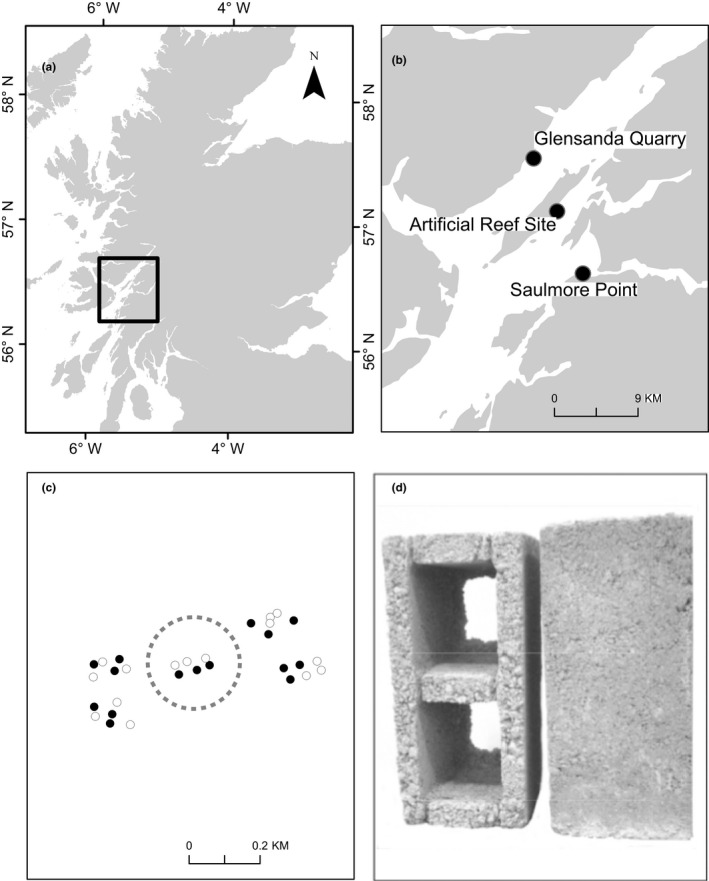
Panel a: Study area on the west coast of Scotland. Panel b: Loch Linnhe, showing location of the artificial reef complex, Glensanda Quarry and Saulmore Point. Panel c: Arrangement of the thirty reef modules into groups/Solid circles represent “simple” modules, open circles represent “complex” modules. The “C” group modules used in this study are showing within the dashed line. Panel d: Two block types used to construct the reef modules: complex blocks (left) and simple block (right)

The Loch Linnhe Artificial Reef complex, on the west coast of Scotland (Figure 1a,b), represents one of the largest reefs in Europe (Sayer & Wilding, 2002). The site comprises thirty individual reef units (“modules”), arranged in groups of six. Environmental characteristics of the reef site (including seabed type, local hydrodynamic regimes) are described in Wilding and Sayer (2002), Wilding (2006), Wilding (2014)

Within each reef group, three of the modules are constructed from “simple” blocks, which are solid concrete blocks (sourced from Glensanda Quarry (Figure 1b)), measuring approximately 21 × 21 × 42 cm. The other three modules are constructed from “complex” blocks, which have two additional voids within them (Figure 1d). The locations of the simple and complex block modules within the group were randomly assigned at the construction phase.

**Figure 2:**
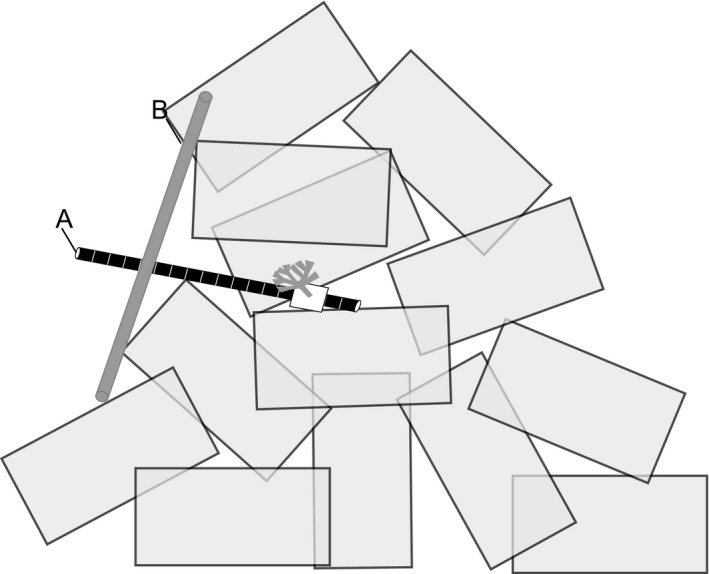
Schematic of experimental setup showing the approximate conical shape of the reef modules. *Flustra foliacea* fronds were secured to pole A at one of four positions. Pole A had a length of either 0.45, 0.9, or 1.20 m. Pole A was inserted into the reef between blocks. Pole B was held perpendicular to pole A as close as possible to the reef edge. The point of intersection between pole A and pole B was recorded as a measure of the distance into the reef

This study was based on the simple and complex block modules of the “C Group” reefs, which are located in 18–21 m of water. In each module, there are approximately 4,000 blocks that form a roughly conical shape (Figure 2). The height of the reef modules varies from 3.7 to 4.9 m (base to tip) (Sayer & Wilding, 2002). Within the C group, modules are between 22 and 52 m apart, and the between‐module distance is smaller between modules of different types (i.e., simple to complex), than between modules of the same type (i.e., complex to complex or simple to simple).

Thirty experimental sites were assigned on each reef module at (predetermined) random heights on the reef, as measured by a digital depth gauges, and (predetermined) random distances around the reef, as measured by the number of times a diver kicked while swimming (“fin kicks”). At each site, a frond of *F. foliacea* was transplanted.

### 2.3 *Flustra foliacea* productivity

*Flustra foliacea* is a marine bryozoan that commonly occurs on a variety of substrata throughout northeast Atlantic subtidal waters. It was selected for this study to quantify the effects of reef design on productivity because of it large colonial form (typically 20–30 cm wide), which can be split into genetically identical segments, thereby reducing the effects of individual differences in growth rates. Furthermore, colonies are robust and able to withstand a range of flow conditions making them amenable to transplantation and manipulative experiments. *F. foliacea* undergoes a single sexual reproduction event, following the growing season, in late autumn, meaning that assessments of productivity could be measured via growth rates and were not confounded by reproduction (Stebbing, 1971). Finally, *F. foliacea* was selected because growth can easily be measured, for example via weight change, area change or increase in zooid numbers (Menon, 1975; O'Dea & Okamura, 2000; Stebbing, 1971).

Twelve colonies of *F. foliacea* were collected from Saulmore Point (Figure 1a), two days prior to the experimental deployment. After collection, colonies were transferred to recirculating aquaria tanks and maintained there for up to 48 hr. Fifteen fronds with no visible epibiont cover were detached by hand from each colony. Each frond was photographed and weighed using a buoyant weight technique, with temperature maintained at 14**°**C and salinity at 33 (Davies, 1989). The buoyant weigh technique enables living samples to be weighed while suspended in seawater. Fronds with a buoyant weight between 1.0 and 1.3 mg were selected to standardize initial frond size. Following removal from the colony, the base of each frond (~1 cm in length) was secured between polypropylene plates. Fronds from the twelve colonies were then randomly assigned to one of the six reef modules. The plates (and frond) were secured to poles to enable them to be transplanted to the reef modules at different distances into the reef structure (Figure 2). A total of 30 poles were used for each module. The poles were either 0.45, 0.90, or 1.20 m in length (10 poles for each of the three length), and were marked with 50 mm intervals.

The plates (with single *F. foliacea* fronds) were secured to the poles at positions of either 0, 0.1, 0.2, 0.3, or 0.4 m from the end of the pole. At each experimental site on the reef, the pole was inserted into the reef, between the reef blocks. A 1.5 m measuring stick was then held perpendicular to the *F. foliacea* pole, and the distance of the pole into the reef was estimated using the marked 50 mm intervals. The position of the frond on the pole (0, 0.1, 0.2, 0.3, or 0.4 m) was then subtracted from the measured distance into the reef to determine the penetration of the *F. foliacea* frond into the reef. This approach allowed frond to be transplanted to a range of depths into the reefs, given that is was not possible to determine how far poles could be inserted before undertaking the transplantation. A schematic of the experimental design is shown in Figure 2.

After 35–40 days deployment, fronds were recovered by SCUBA diver and placed in recirculating aquaria tanks. At all times prior to, and after, deployment fronds were maintained in seawater. Recovered fronds were assessed for damaged by comparison with predeployment photographs. Damaged fronds were discarded. Undamaged fronds were reweighed using the buoyant weight technique. The change in weight was divided by the weight of the initial frond to give a relative growth rate (mg/g). The relative growth rate was then divided by the number of weeks that each frond was deployed for to give a metric of mg.g^‐1^.week^‐1^, referred to henceforth as productivity rate.

### 2.4 Data analysis

Initial data exploration was carried out prior to analysis to check for outliers, homogeneity of variance, colinearity of variables, and obvious trends in the response variable using a visual assessment of exploratory plots, model diagnostic plots, and draftsman plots (Zuur, Ieno, & Elphick, 2010). A normal mixed‐effects linear model was fitted to the data with productivity rates of *F. foliacea* fronds (log transformed) as the response variable. There were two fixed predictor variables in the model: block type (simple and complex) and distance into the reef (0–1.2 m). There were two random sources of variance in the experimental design: module (six levels) and colony (twelve levels).

The optimal model of productivity rates was selected from candidate models following a backward selection process (Zuur, Ieno, Walker, Saveliev, & Smith, 2009). The decision to include terms was based on assessing the model with and without variables using Akaike's Information Criterion (AIC) and a likelihood ratio test. The selection process was first carried out on the random effects, conditional on all fixed effects and interactions. Where random effects did not improve the model fit, they were excluded (Korner‐Nievergelt et al., 2015). Once optimized in terms of the random effects, the model was further simplified using a backwards selection procedure, to drop fixed effect terms (starting with interactions) and assessing model fit using the AIC and likelihood ratio test. This process was repeated for all fixed terms until the most parsimonious (optimal) model had been identified.

Following an initial maximum likelihood model fit to the data, Bayesian inference, with noninformative priors, was used to obtain parameter estimates and the associated uncertainty (Korner‐Nievergelt et al., 2015). Bayesian inference allowed for the probabilities of different models (hypotheses) of *F. foliacea* productivity (given the data) to be calculated. The maximum likelihood parameter estimates were used to describe the joint posterior distribution, which specifies combinations of parameter values that are plausible, given the data. From the joint posterior distribution, 2000 possible model parameters were drawn (simulated) using the function “sim” in the R package “arm” (Gelman & Yu‐Sung, 2015). The mean of the simulated values was used as the final parameter estimate and the 2.5% and 97.5% quantiles used to represent the lower and upper limits of the 95% credible intervals (CrI), within which there is a 95% certainty that the true parameter lies. The probabilities of different models (hypotheses) of *F. foliacea* productivity (given the data) were obtained by calculating the number of simulations that met each condition. All statistical analysis was done using R version 3.0.0; mixed‐effect models were developed using the R “nlme” and “lme4” libraries (Bates, Mächler, Bolker, & Walker, 2014).

## 3 RESULTS

**Figure 3:**
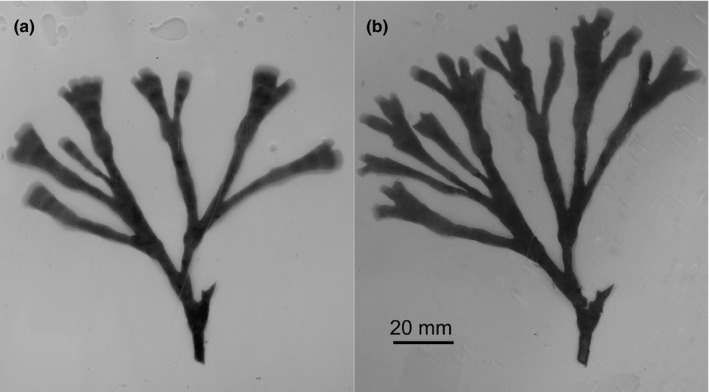
An example of a frond of *Flustra foliacea* before (a) and after (b) deployment to a complex module of the Loch Linnhe Artificial Reef

Of the 180 fronds deployed to the Loch Linnhe Artificial Reef, 62% were recovered undamaged. Fronds were observed to be actively feeding (lophophores extended and visible to the naked eye) prior to recovery, and epibiont growth was not visible on any of the recovered fronds. An example of a frond before and after deployment is shown in Figure 3a. The average productivity rate of fronds across all reef modules was 747 mg g^−1^ week^−1^ (639–855, 95% CrI).

Both of the random factors “colony” and “module” only explained a small portion (<1%) of the total variance, and including them did not substantially improve the model fit (Colony: ΔAIC = 0.04, *L* = 0.431, *p* = .153; Module: ΔAIC = 1.57, *L* = 0.428, *p* = .513). The final model, therefore, did not include any random effects. The optimal model for *F. foliacea* productivity rates on the Loch Linnhe Artificial Reef included the following fixed effects: block type, distance into the reef and the interaction of block type and distance into the reef. Inclusion of the interaction term significantly improved model fit (ΔAIC = 16.0, *F* = 19.3, *p* = <.001).

**Figure 4:**
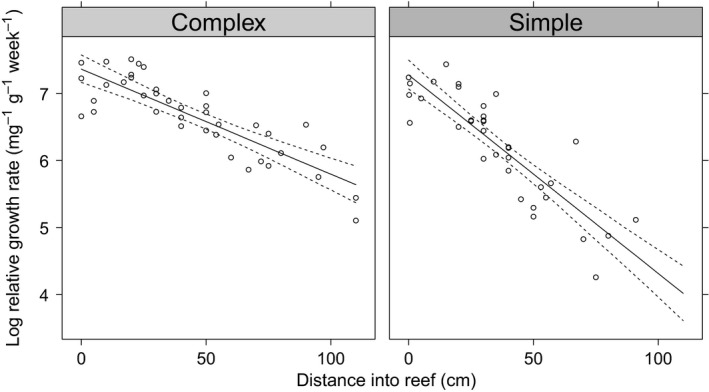
Modeled relationship and 95% CrI interval between productivity and distance into the reef for *Flustra foliacea* fronds transplanted on to the simple and complex modules of the C group of the Loch Linnhe Artificial Reef. Circles represent raw data points

The modeled productivity rates were on average 2.4 times (1.6–3.5, 95% CrI) higher on complex modules compared with simple modules (Table 1). The regression lines describing the relationship between productivity rates and distance into the reef with 95% credible intervals are shown in Figure 4. For both reef types, the productivity rates decreased with distance into the reef, but the rate of decrease varied according to the block type (Figure 4 and Table 1), with a decrease of 1.56% (1.19–1.93, 95% CrI) for every 1 cm into the reef on complex modules and a decrease of 2.93% per cm (1.96 = 3.88, 95% CrI) on simple modules. At the edge of the reef (0 m), there was a little difference in the productivity rates of fronds (1.07 higher,(0.719–1.67, 95% CrI) on complex reefs than on simple reefs), whereas 1 m into the reefs, productivity rates were 4.4 times higher (2.46–8.08, 95% CrI) on complex reefs than on simple reefs. The probability (given the data) that productivity rates were higher on complex reefs than on simple reefs was >.999, and the probability that distance into the reef had a negative effect on productivity rates was >.999.

**Table 1:** Estimates of the effect of reef type (simple vs. complex) and distance into the reef (DIR) on log *Flustra foliacea* productivity rates

Parameter	Estimate (mean)	2.5%	97.5%
(Intercept)	7.36	7.16	7.57
Simple Reefs	−0.0827	−0.379	0.188
DIR	−0.0157	−0.0195	−0.0120
Simple:DIR	−0.0140	−0.201	−0.00779

Note

The 2.5% and 97.5% quantiles represent the 95% credible interval around the parameter estimates.

## 4 DISCUSSION

Parameters for production calculations (e.g., density, relative growth rates, mortality, behavior, and diets) are necessary to specify empirical models of the productive capacity of structures in the marine environment (Dolbeth et al., 2012), and to resolve the uncertainty associated with artificial reefs as mitigation (Powers, Grabowski, Peterson, & Lindberg, 2003). Understanding the growth rates of sessile epifauna, their contribution to artificial reef secondary productivity, and energy transfer pathways in reef communities will facilitate more accurate models of reef productivity and predictions of the biomass of commercial species associated with structures (Svane & Petersen, 2001).

On the Loch Linnhe Artificial Reef, productivity rates of *F. foliacea* varied according to location on reef modules and between reef modules constructed from different block types. Previous estimates of the productivity capacity of structures have been based on measurements of the structure's external surface area, and assumed that all surfaces of the structure contribute equally to the productive output (Figley, 2003). The results obtained during this study show that the contribution of different reef areas to the total productive output can vary markedly. The results also show that internal areas of a reef contribute to the total productive output of a structure and do not function as unproductive structural components.

Distance into the reef module and the type of reef were both important factors in determining the productivity of transplanted fronds. Moving away from the reef edge to the internal areas, it would be expected that water motion and the subsequent delivery of food to suspension feeders would decrease, since the structure would baffle or redirect currents (Liu & Su, 2013; Walker, Schlacher, & Schlacher‐Hoenlinger, 2007). As both current velocity and food availability are known to drive growth rates of suspension feeders (Wildish & Kristmanson, 2005), the decrease in *F. foliacea* productivity toward the center of the reef modules is likely to be a consequence of a reduction in these two factors. The two block types used to construct the Loch Linnhe Artificial Reef give rise to differences in the level of habitat complexity at scales that are relevant to benthic suspension feeders (Hunter & Sayer, 2009). It has been suggested that increased habitat complexity (at the scale of the whole reef) influences total reef productivity because of the greater surface area and greater range of niches available on more complex reefs (Jacobi & Langevin, 1996). On the Loch Linnhe Artificial Reef, it is less likely the differences in surface area, mediated by habitat complexity, between simple and complex modules, would influence the productivity of singular fronds, since the surface of the individual block types (to which fronds were transplanted) is equally complex. This is supported by the similarity of *F. foliacea* growth rates obtained at the reef edge (i.e., 0 m into the reef—Figure 4) for both reef types. It is more likely that the observed differences in habitat complexity between simple and complex modules led to differences in the porosity of the reefs, and subsequently the water flows and food supply to the internal areas of the structures (Enochs, Toth, Brandtneris, Afflerbach, & Manzello, 2011). Given that simple blocks have a larger surface area, which will baffle currents, it would be expected that there would be a sharper decrease in water flow toward the center of the reef for simple modules than for complex modules. Again, any difference in water motion and food supply of internal areas resulting from the block type could explain why the productivity rates of fronds 1 m into reef were substantially less on simple modules compared with complex modules. Coupling productivity rates of transplants with experimental measures of the internal flow conditions and food supply would be challenging due to the limited access to the internal reef surfaces, and the need for sufficiently small flow meters and sediment traps that could be deployed between reef blocks and recovered without disturbance. Computational fluid dynamic simulations of the internal flow fields on the Loch Linnhe Artificial Reef (e.g., as per Liu, Guan, Zhao, Cui, and Dong (2012)) present a method for quantifying the exact relationship between productivity rates and hydrodynamic regimes, as mediated by the reef design. Regardless of the limitations in measuring the drivers of the different productivity rates, our results suggest that introducing voids to reef blocks may allow more of the internal surface areas to become functional habitat for suspension feeders and could lead to an overall increase in the productivity capacity.

In addition to the physical drivers of secondary production (such as those explored in this study), trophic interactions play an important role in the structure and function of artificial reef communities. Langhamer (2012) and Eklund (1997) demonstrated the role of predation in driving the diversity, abundance, and production of artificial reef fauna. On natural reef habitats, O'Gorman, Enright, and Emmerson (2008) showed that increased top predator diversity led to increased secondary production within a temperate marine food web, while Eklund (1997) found that predation on artificial reefs was more important than provision of benthic food resources in determining fish production. However, settlement panel experiments by Freestone, Osman, Ruiz, and Torchin (2011) suggest that there is a strong latitudinal gradient in the role of predation in structuring epifauna communities, with minimal to no effect of predation on epifauna species richness in temperate waters. In the present study, the role of biotic factors in driving secondary productivity was not quantified. Furthermore, patterns of secondary productivity were inferred from a single indicator species, and they may not necessarily reflect the productivity patterns of all filter‐feeding organisms, which may have different sensitivities and habitat requirements. Additional research that incorporates a broader range of reef‐dwelling organisms is required to elucidate intraspecies variations and the relative importance of different abiotic and biotic factors in driving secondary productivity on the Loch Linnhe Reef.

Generating data on the spatial variation in epifaunal productivity rates on artificial reefs (such as the data provided by this study) is the first stage in efforts to incorporate biological knowledge of functional habitat into reef productivity models. The results obtained in this study suggest that total surface area of reef should not be used to model the productive capacity of artificial reefs, and instead, the effective productive area must be calculated. With such information, it also becomes possible to suggest modifications to the design of infrastructure (e.g., introduce voids to scour protection material) to achieve particular management goals. This could improve the positive environmental impacts of structures and potentially increase their capacity to serve as mitigation, for example for loss of downstream habitat.

## CONFLICT OF INTEREST

None declared.

## AUTHOR CONTRIBUTIONS

SR, TW and, JP coconceived the research questions, approach, and methodology. SR undertook the majority of the data acquisitions, with contributions from TW and JP. SR undertook data analysis and initial drafting of the manuscript. JP and TW critically reviewed the manuscript and gave final approval for publication.

## Data Availability

Sampling locations (latitude, longitude, and depth) and Flustra mass data are available as Supporting information.
